# A Comparison of the Use of Smart Devices, Apps, and Social Media Between Adults With and Without Hearing Impairment: Cross-sectional Web-Based Study

**DOI:** 10.2196/27599

**Published:** 2021-12-20

**Authors:** Marieke F van Wier, Emily Urry, Birgit I Lissenberg-Witte, Sophia E Kramer

**Affiliations:** 1 Ear & Hearing, Otolaryngology-Head and Neck Surgery Amsterdam Public Health research institute Amsterdam UMC, Vrije Universiteit Amsterdam Amsterdam Netherlands; 2 Research & Development Sonova AG Stäfa Switzerland; 3 Epidemiology and Data Science Amsterdam UMC, Vrije Universiteit Amsterdam Amsterdam Netherlands

**Keywords:** hearing impairment, social media use, app use, benefits from social media, eHealth, mobile phone

## Abstract

**Background:**

eHealth and social media could be of particular benefit to adults with hearing impairment, but it is unknown whether their use of smart devices, apps, and social media is similar to that of the general population.

**Objective:**

Our aim is to study whether adults with normal hearing and those with impaired hearing differ in their weekly use of smart devices, apps, and social media; reasons for using social media; and benefits from using social media.

**Methods:**

We used data from a Dutch cohort, the National Longitudinal Study on Hearing. Data were collected from September 2016 to April 2020 using a web-based questionnaire and speech-in-noise test. The results from this test were used to categorize normal hearing and hearing impairment. Outcomes were compared using (multiple) logistic regression models.

**Results:**

Adults with impaired hearing (n=384) did not differ from normal hearing adults (n=341) in their use of a smartphone or tablet. They were less likely to make use of social media apps on a smartphone, tablet, or smartwatch (age-adjusted odds ratio [OR] 0.67, 95% CI 0.48-0.92; *P*=.02). Use of social media on all devices and use of other apps did not differ. Adults with hearing impairment were more likely to agree with using social media to stay in touch with family members (OR 1.54, 95% CI 1.16-2.07; *P*=.003) and friends (age-adjusted OR 1.35, 95% CI 1.01-1.81; *P*=.046). Furthermore, they were more likely to agree with using social media to perform their work (age-adjusted OR 1.51, 95% CI 1.04-2.18; *P*=.03). There were no differences in the experienced benefits from social media.

**Conclusions:**

The potential for eHealth is confirmed because adults with hearing impairment are not less likely to use smart devices than their normal hearing peers. Adults with hearing impairment are less likely to use social media apps on a smart device but not less likely to use social media on all types of internet-connected devices. This warrants further research on the types of social media platforms that adults with hearing impairment use and on the type of device on which they prefer to use social media. Given that participants with hearing impairment are more likely than their normal hearing peers to use social media to perform their work, use of social media may be seen as an opportunity to enhance vocational rehabilitation services for persons with hearing impairment.

## Introduction

### Background

Disabling hearing loss affects 466 million people worldwide [1]. Its prevalence will double by 2050 because of increasing life expectancy. Hearing impairment is one of the most prevalent disabilities because hearing deteriorates with age. Almost one-third of people aged ≥65 years have disabling hearing loss [1]. However, because age-related hearing loss can start earlier, a large number of middle-aged adults also have hearing disabilities. In high-income countries, 15%-25% of adults between the ages of 50 and 65 years have mild to severe hearing loss [2,3]. A recent study in the Netherlands estimated that 1.2 million adults aged ≥40 years, or 13% of the population in that age bracket, have disabling hearing loss [4]. Hearing difficulties can be mitigated by the use of communication strategies, speech reading, hearing aids, and hearing assistive technology. A smaller number of people with impaired hearing, mostly those who have been deaf or hard of hearing from a young age, communicate in sign language as their native language.

Most adults in high-income countries have access to the internet, although its use is less ubiquitous in older adults [5,6]. This gives potential for digitalized hearing health care, both stand-alone and adjunct to in-person care [7-9]. Digital hearing health care facilitates patient-centered care in the comfort of one’s own home. In this way, next to making hearing health care more accessible, digital care could perhaps boost the lagging uptake of communication strategies and hearing aids [10]. The current COVID-19 pandemic has sped up the shift to remote audiological care, although audiologists still have concerns about patients’ access to technology and their preferences [11]. During the UK lockdown, teleaudiology was particularly used for (tinnitus) counseling [11]. The internet offers many synchronous and asynchronous communication options that support counseling, for example, email, direct messaging, social network sites and apps, and video calling. With the development of web-based hearing assessment and hearing aid fitting, these in-person services will also be offered on the web [12]. In addition to teleaudiology, internet-mediated communication could also support psychosocial health by fostering social connection. Mild to severe hearing loss makes it difficult to follow conversations in certain situations, even with the use of a hearing aid, leading to less meaningful interactions and withdrawal from social activities [13]. Loneliness, depression, and anxiety are more prevalent in adults with hearing loss than in their peers who have no hearing problems [13-16]. Communication and connection with others through the internet could replace some of the face-to-face contacts and mitigate these negative outcomes [17]. This raises the question of whether adults with hearing impairment use the internet, smart devices, apps, and social media as much and for the same reasons as their normal hearing peers and the general public. If so, it would be reasonable to move (some) services to the web, which would substantiate the opportunities for social support. Earlier research disputes the assumption of equal use.

So far, only 3 studies have looked at internet access and use among adults with hearing impairment in high-income countries. Of these studies, 2 [18,19] showed that older adults with hearing difficulty were less likely to use the internet, whereas the other [20] found no differences between users of hearing aids and the general population overall. However, the latter study did find a higher internet use among users of hearing aids aged ≥75 years compared with the (age-matched) general population. Thus, there is inconclusive evidence regarding whether adults with hearing loss make different use of the internet than their normal hearing peers. Moreover, these studies were performed at a time when the internet was mostly accessed through desktop computers and was less pervasive in daily life. A more recent marketing survey looked at whether ownership and use of a smartphone is different in adults with a hearing impairment [21]. This 2018 UK survey found that the rates of smartphone ownership were 53% in people with self-reported hearing impairment and 81% in people who reported having no disabilities. Furthermore, 44% of the adults with hearing impairment used their mobile device to access the internet, whereas this rate was 69% in respondents without disabilities. However, 60% of the people who reported a hearing impairment were aged >65 years compared with only 16% of the nondisabled people. The results might therefore reflect the differences in age between both groups. The same survey found that people with a hearing impairment had quite similar reasons for using the internet as the people who reported no disability. Comparable results were obtained from a 2016 US survey [22]. This survey asked people with self-reported hearing disabilities which features or functions they use on their smartphone. Use of the internet in general and of social media apps in particular seems to be a little lower in people with a hearing disability compared with the general population, but as in the UK survey, this may also reflect differences in age.

Several studies have analyzed social media use by distinct groups of adults with hearing impairment by extracting publicly available content and user data (manual and automated *web scraping*) posted on social network sites such as Facebook, Twitter, and YouTube, as well as from web-based forums and from personal blogs [23-28]. This type of research gives insight into the information that is shared, the feelings and opinions of the posters, and the nature of communication on these platforms, but there are also limitations. Content analysis studies do not give insight into either the relative frequency of social media use or its passive use (ie, reading and watching). Furthermore, the characteristics of the posters, such as age, sex, income, education level, and whether they have hearing loss and to what extent, are unknown or not verifiable. This impedes specification of who uses which type of social media and in which way and who does not. In addition, there is no (legal) access to private communication such as WhatsApp messages and private Facebook pages. Postings on social media also do not provide information about the potential benefits of using social media, for example, whether it has strengthened the social connections of the user. In conclusion, these studies do not give insight into the social media use of all adults with hearing impairment: whether they use social media for private communication, the extent of use, and their reasons for using social media; the experienced benefits from using social media; and whether all these data are comparable with those found in the general public and normal hearing adults. The latter would justify relying on data from the general public when making decisions on using computer-mediated communication with adults with hearing impairment. We did not find studies that addressed these questions.

### Research Questions

This study therefore investigated the following research questions (RQs):

- RQ1: Do adults with normal hearing and those with impaired hearing differ in the use of smart devices?
- RQ2: Do adults with normal hearing and those with impaired hearing differ in the use of different types of apps?
- RQ3: Do adults with normal hearing and those with impaired hearing differ in the use of social media?
- RQ4: Do adults with normal hearing and those with impaired hearing differ in their reasons for using social media?
- RQ5: Do adults with normal hearing and those with impaired hearing differ in the experienced benefits from using social media?

## Methods

### Overview

Data were available from a long-running Dutch national cohort of adults with normal hearing and those with impaired hearing, the National Longitudinal Study on Hearing (NL-SH). Demographics and use of technology, apps, and social media were collected through a web-based questionnaire. The hearing status of participants was determined by a web-based speech-in-noise test.

### Details of NL-SH

#### Recruitment and Measurements

Initiated in 2006, the NL-SH is an ongoing, prospective cohort study in which both adults with normal hearing and those with impaired hearing participate. This cohort was set up to gain knowledge on the long-term trajectory of hearing loss and its association with psychosocial health, work outcomes, and health care use in adults of working age. The NL-SH uses convenience sampling. The major portals for recruitment are the web-based Dutch National Hearing Test (NHT) [29] and the study website [30] where the public can take the same hearing test. It offers the general public a fast and convenient way to test their own ability to recognize speech in noise (further described in the *Speech-in-Noise Test* section). After presenting the test results, they are asked if they are interested in taking part in hearing-related research. If they are interested, they are taken to the introduction page of the NL-SH on the study website [30], where the study is fully explained and they can download an information brochure. Prospective participants can then choose to enroll. At enrollment, sex, age, and contact details (email address, home address, and phone number) are asked. Age is checked against the inclusion criterion of being 18-70 years; those who do not fit this criterion cannot enroll. Other eligibility criteria are not set for the NL-SH. Those who are eligible have to take a speech-in-noise test specific to the NL-SH and are sent a link to the web-based study questionnaire.

Inclusion measurements (T0) started in 2006 and still continue. The 5-year follow-up (T1) started in 2011, and the 10-year follow-up (T2) started in 2016. For this study, only the T2 measurements were used. The invitation for the T2 measurement round was sent approximately 10 years after the T0 hearing test was performed. In all, 2 email reminders and a postal reminder were sent within a period of 3 months after the first invitation to fill out the questionnaire. In general, the T2 hearing test is performed directly after participants fill out the web-based questionnaire, but a delay of up to 3 months is possible. People who did not perform this test received a total of 3 email reminders during these 3 months. To be included in this study, both the T2 questionnaire and hearing test had to be fully completed. Data collected up to April 1, 2020, were included in the analyses.

The NL-SH study protocol has been approved by the Medical Ethics Committee of the Amsterdam Medical Center, location VUmc, in Amsterdam, the Netherlands (METC number: 2006/83; ToetsingOnline NL12015.029.06).

#### Questionnaire

The questionnaire consisted of questions on demographics; hearing; use of technology, apps, and social media; and reasons for using social media and benefits of this use. The questions on use of technology and apps were taken from a web-based marketing study (Sonova AG, written communication, 2019). The validity of these questions is unknown. Standardized questionnaires about use of social media, reasons for using social media, and the experienced benefits from using social media are not available. The questions on these topics were devised by the research team and pilot-tested in a group of adults with hearing impairment. Textbox 1 lists the questions other than demographics. The full questionnaire can be found in Multimedia Appendix 1.

**Overview of the questions on use of technology, apps, and social media, as well as reasons for using social media and benefits of social media use.**

**Questions and answer options used in this study**

- Which of these devices do you use at least once a week?
  Smartphone
  Smartwatch
  Tablet
- Which types of apps do you use at least once a week on your current smartphone, tablet, or smartwatch?
  Weather
  News
  Finances (mobile banking, stock exchange, etc)
  Navigation
  Remote control (television, stereo system, etc)
  Fitness
  Communication (email, WhatsApp, WeChat, etc)
  Medical or health
  Social media (Facebook, Instagram, Twitter, etc)
  Music and podcasts
- Do you use social media?
  Yes
  No
- To what extent do you agree with the following statements (rated on a scale of 1-10: fully disagree=1, fully agree=10)? I use social media to...
  Stay in touch with family members
  Stay in touch with acquaintances
  Stay in touch with colleagues or peers
  Share experiences, videos, or photos
  View experiences, videos, or photos
  Expand my work-related network
  Perform my work
  Gain new knowledge
  File complaints and problems with the government or businesses
- With what frequency do you use social media?
  Multiple times a day
  Daily
  Weekly
  Monthly
  A couple of times a year
- What have you gained from your social media use so far?
  New acquaintances
  New friendships
  Closer or more intense family ties
  Closer or more intense friendships
  Expanded work-related network
  New knowledge about health
  I have gained little or nothing from social media

Demographics concerned sex, current age, highest attained education, and first language Dutch or other. Education was divided into low (elementary school or attended high school but no degree), medium (high school graduate or having an associate’s degree), and high (having a bachelor’s degree, master’s degree, or doctoral degree). In all, 2 hearing-related questions asked about having normal hearing or some type of hearing loss and, for those with self-reported hearing loss, whether they use a hearing aid.

Use of technology consisted of a list of devices with the question “Which of these devices do you use at least once a week?” The list included devices such as a traditional mobile phone, smartphone, traditional wristwatch, smartwatch (eg, Apple watch), fitness watch (eg, Fitbit), laptop or notebook computer, tablet, television, radio, and more. The list did not mention a PC. Participants could tick the box for all devices that applied. For this study, the use of only mobile devices on which an app can be installed (ie, a smartphone, a tablet, or a smartwatch), further referred to as a smart device, was analyzed.

Next, participants who indicated that they used a smart device were asked what types of apps they used at least once a week. The options to tick were as follows: weather, news, finances (mobile banking, stock exchange, etc), navigation, remote control (television, stereo system, etc), fitness, communication (email, WhatsApp, WeChat, etc), medical or health, social media (Facebook, Instagram, Twitter, etc), music or podcasts, and other. The category *other* was not included in the analyses.

Participants were then asked if they use social media, for example, Facebook, LinkedIn, Skype, WhatsApp, Twitter, and Instagram. If they said “yes” or if they had a missing answer on this question, they were asked about frequency of use (1 answer possible): multiple times a day, daily, weekly, monthly, or a couple of times a year. In the analyses, monthly and a couple of times a year were collapsed into 1 category. Those who used social media were asked how much they agreed with several statements about reasons for using social media. The reasons provided were as follows: to stay in touch with family; to stay in touch with friends; to stay in touch with acquaintances; to stay in touch with colleagues or peers; to share experiences, videos, or photos; to view experiences, videos, or photos; to expand work-related network; to perform work; to gain new knowledge; and to file complaints. These questions could be answered on a scale from 0 to 10 (an 11-point Likert scale), with 0=*fully disagree* (coded as 1), 5=*do not agree, do not disagree* (coded as 6), and 10=*fully agree* (coded as 11), resulting in scores of 1-11, to be handled as a continuous outcome.

Finally, social media users were asked what they had gained from social media use so far. They could choose any of the following answers: new acquaintances, new friendships, closer or more intense family ties, closer or more intense friendships, expanded work-related network, new knowledge about health, gained little or nothing, and other. The category *other* was not used in the analyses.

At the end of the questionnaire, a personal link was provided to the web-based speech-in-noise test.

#### Speech-in-Noise Test

The ability to recognize speech in noise is salient for measuring the disabling effects of hearing impairment because one of the first and major complaints of adults with hearing impairment is difficulty in understanding what is said when there is background noise [31]. To measure speech-in-noise recognition, the procedures of the NHT were followed. First developed for use by phone, the internet version of the NHT was launched in 2005. To ensure comparability to the earlier measurements, the procedures of this original version are still used in the NL-SH measurements. Participants are instructed to perform the test in a quiet room. Users of hearing aids are instructed to perform the test without their hearing aids. All participants are asked to use headphones for the test, but speakers are also allowed. Participants have to indicate which transducer they used. The test is binaural (ie, diotic), and the results are mainly representative of the better ear. A total of 23 digit triplets (eg, 6-2-5) are presented against a background of masking noise in an adaptive manner: the noise level is fixed in the test, and the speech level varies. After each incorrect response, the subsequent triplet is presented at a level higher by 2 dB, increasing the signal-to-noise ratio (SNR) a level higher of 2 dB. If the participant provides a correct response, the subsequent triplet is presented at a level lower by 2 dB. The speech-reception threshold in noise (SRTn) is calculated by taking the average SNR of the last 20 presentations, corresponding to a score of 50% of the presented triplets understood correctly. Because of the design of the test, the SRTn values range from –13.4 dB SNR to a ceiling level of 4 dB SNR.

Validation of the original NHT version showed a high correlation (ρ=0.866) with SRTns derived from the standard test in the Netherlands that uses sentences in stationary speech-shaped noise. The measurement error (SE of measurement) is estimated to be <1 dB [32]. Compared with the standard sentence-in-noise test, the NHT phone version has a sensitivity of 0.91 and a specificity of 0.93 at a cutoff of –4.1 dB for hearing impairment [32]. Because of the benefit of listening with 2 ears, the cutoff of the internet version was adjusted with 1.4 dB [33]. This gives a cutoff of –5.5 dB to divide the group into adults with normal hearing ability to recognize speech in noise (further indicated as normal hearing) and those with impaired hearing. It should be taken into account that diotic speech understanding in noise is less compromised in conductive and mixed hearing losses. This means that participants with these types of hearing loss, even those normally using a hearing aid, may be classified as normal hearing. Participants using speakers may perform slightly worse in the test than if they had used headphones, resulting in misclassification to the group with hearing impairment for some of the participants with normal hearing.

### Statistical Analysis

Normally distributed continuous data are described with means and SDs, whereas nonnormally distributed continuous data are described with medians and IQRs. For nominal and ordinal data, frequencies are reported.

Adults with impaired hearing and normal hearing adults were compared using multiple logistic regression analysis. Age, sex, and education level were considered potential confounders. They were included in the model if (1) the potential confounder had influence (*P*<.10) on both the outcome and the independent variable and (2) the regression coefficient of the influencing factor changed by ≥10% after adding the potential confounder to the model.

Answers to the items that were scored on an 11-point Likert scale (fully disagree-fully agree) were not normally distributed and had to be categorized. To prevent uneven numbers in the categories, they were categorized into 3 groups based on approximate 1/3 divisions with increasing levels of agreement. Multiple ordinal logistic regression analysis was used to analyze whether the distribution of these answers and answers about the frequency of social media use (several times a day, daily, weekly, monthly, or a couple of times a year) differed between adults with normal hearing and those with hearing impairment. Assumptions of logistic analysis were tested in all analyses. For the ordinal results where the assumption of proportional odds was not met, multinomial logistic regression analysis was used. The results were considered statistically significant if *P*<.05. Analyses were conducted using SPSS software (base edition with custom tables and advanced statistics add-on; version 26.0; IBM Corporation).

## Results

### Overview

A total of 885 study participants responded to the T2 measurement round between September 15, 2016, and April 1, 2020. Of the 885 participants, 837 (94.6%) fully filled out the questionnaire. Of these 837 participants, 725 (86.6%) also performed the speech-in-noise test. Of the 112 participants who did not perform the speech-in-noise test, 68 (60.7%) reported in the questionnaire that they had hearing loss.

Of the 725 participants with complete data, 619 (85.3%) participated from September 2016 to December 2017. Most of the participants were women (442/725, 61%). The participants’ mean age was 57.7 (SD 11.4) years, and 60% (435/725) had a high level of education (Table 1).

Of the total group, 53% (384/725) had a hearing test score ≥–5.5 dB SNR and were subsequently classified as having a hearing impairment. Table 1 shows the characteristics of these participants and of the normal hearing participants separately.

**Table 1:** Overview of characteristics and categorical outcomes for the total group, a group with normal hearing, and a group with hearing impairment (N=725).

Characteristics		Total group (N=725)	Normal hearing (n=341)	Hearing impairment (n=384)
Female, n (%)		442 (61)	200 (58.7)	242 (63)
Age (years), mean (SD)		57.7 (11.4)	55.5 (10.9)	59.6 (11.4)
**Education level, n (%)**				
	Low	92 (12.7)	39 (11.4)	53 (13.8)
	Medium	198 (27.3)	96 (28.2)	102 (26.6)
	High	435 (60)	206 (60.4)	229 (59.6)
First language (Dutch), n (%)		703^a^ (98.6)	331 (99.1)	372 (98.1)
Self-reported hearing impairment, n (%)		428 (59)	109 (31.9)	319 (83.1)
Hearing aid use, n (%)		299 (41.2)	46 (13.5)	253 (65.9)
Test with headphones, n (%)		319 (44)	211 (61.9)	108 (28.1)
**Weekly use of smart devices, n (%)**				
	Smartphone	612 (84.4)	298 (87.4)	314 (81.8)
	Tablet	420 (57.9)	196 (57.5)	224 (58.3)
	Smartwatch	18 (2.5)	8 (2.3)	10 (2.6)
**Weekly use of apps,^b^ n (%)**		660 (100)	313 (100)	374 (100)
	Weather	456 (69.1)	217 (69.3)	239 (68.9)
	News	453 (68.6)	210 (67.1)	243 (70)
	Financial	222 (33.6)	103 (32.9)	119 (34.3)
	Navigation	283 (42.9)	132 (42.2)	151 (43.5)
	Remote control	148 (22.4)	71 (22.7)	77 (22.2)
	Fitness	59 (8.9)	33 (10.5)	26 (7.5)
	Communication	551 (83.5)	362 (83.7)	289 (83.3)
	Medical or health	82 (12.4)	45 (14.4)	37 (10.7)
	Social media	374 (56.7)	198 (63.3)	176 (50.7)
	Music and podcasts	175 (26.5)	97 (30.9)	78 (22.5)
I use social media, n (%)		625^c^ (86.6)	302 (88.6)	323^d^ (84.8)
**Frequency of social media use, n (%)**		627 (100)	302 (100)	325 (100)
	Multiple times a day	310 (49.4)	149 (49.3)	161 (49.5)
	Daily	241 (38.4)	118 (39.1)	123 (37.8)
	Weekly	56 (8.9)	27 (8.9)	29 (8.9)
	Monthly or a couple of times a year	20 (3.2)	8 (2.7)	12 (3.7)
**Experienced benefits from social media use, n (%)**		628 (100)	302 (100)	326 (100)
	New acquaintances	114 (18.2)	59 (19.5)	55 (16.9)
	New friendships	72 (11.5)	37 (12.3)	35 (10.7)
	Closer or more intense family ties	202 (32.2)	89 (29.5)	113 (34.7)
	Closer or more intense friendships	161 (25.6)	78 (25.8)	83 (25.5)
	Expanded work-related network	144 (22.9)	81 (26.8)	63 (19.3)
	New knowledge about health	141 (22.5)	58 (19.2)	83 (25.5)
	Little or no benefit from social media	184 (29.3)	97 (32.1)	87 (26.7)

^a^n=713.

^b^Use of an app on a smartphone, tablet, or smartwatch.

^c^n=722.

^d^n=384.

### RQ1: Weekly Use of Smart Devices

Most of the participants made weekly use of a smartphone: 87.4% (298/341) of the normal hearing group and 81.8% (314/384) of the group with impaired hearing (Table 1). Logistic regression analysis, which had to be adjusted for age, revealed that there was no statistically significant difference between the 2 groups in the weekly use of a smartphone (odds ratio [OR] 0.82, 95% CI 0.53-1.25; *P*=.35; Table 2).

**Table 2:** Odds ratios (ORs) for smart device use, social media use, and experienced benefits from social media use for adults with hearing impairment compared with normal hearing adults; results from (multiple) logistic regression analysis.

Outcome			Crude model				Age-adjusted model^a^		
			OR (95% CI)		*P* value		OR (95% CI)		*P* value
**Weekly use of smart devices**									
	Smartphone	0.65 (0.43-0.977)		.04		0.82 (0.53-1.25)		.35	
	Tablet	1.04 (0.77-1.39)		.82		N/A^b^		N/A	
**Weekly use of apps^c^**									
	Weather	0.98 (0.70-1.36)		.90		0.90 (0.63-1.25)		.50	
	News	1.15 (0.83-1.59)		.42		N/A		N/A	
	Financial	1.06 (0.77-1.47)		.71		N/A		N/A	
	Navigation	1.06 (0.78-1.44)		.73		N/A		N/A	
	Remote control	0.97 (0.67-1.40)		.88		0.91 (0.63-1.33)		.62	
	Fitness	0.69 (0.40-1.18)		.17		0.79 (0.45-1.36)		.39	
	Communication	0.97 (0.64-1.46)		.88		0.99 (0.65-1.52)		.98	
	Medical or health	0.71 (0.45-1.13)		.15		N/A		N/A	
	Social media	0.60 (0.44-0.82)		.001		0.67 (0.48-0.92)		.02	
	Music and podcasts	0.65 (0.46-0.91)		.01		0.73 (0.51-1.05)		.09	
I use social media			0.72 (0.47-1.11)		.14		0.90 (0.57-1.42)		.65
**Experienced benefits from social media use**									
	New acquaintances	0.84 (0.56-1.26)		.39		N/A		N/A	
	New friendships	0.86 (0.53-1.41)		.55		0.93 (0.56-1.54)		.78	
	Closer or more intense family ties	1.27 (0.91-1.78)		.16		1.21 (0.86-1.71)		.28	
	Closer or more intense friendships	0.98 (0.69-1.40)		.92		1.10 (0.76-1.59)		.62	
	Expanded work-related network	0.65 (0.45-0.95)		.03		0.78 (0.53-1.14)		.20	
	New knowledge about health	1.44 (0.98-2.10)		.06		N/A		N/A	
	Little or no benefit from social media	0.77 (0.55-1.09)		.14		0.72 (0.51-1.03)		.07	

^a^Age in quartiles: Q1: 29-49 years; Q2: 50-59 years; Q3: 60-66 years; and Q4: 67-81 years.

^b^N/A: not applicable; no age adjustment necessary.

^c^Use of an app on a smartphone, tablet, or smartwatch.

The rates of weekly use of a tablet were 57.5% (196/341) in the normal hearing group and 58.3% (224/384) in the group with hearing impairment. Logistic regression analysis showed that these percentages did not differ between the groups (OR 1.3, 95% CI 0.77-1.4; *P*=.82; Table 2).

Because of the small number of people using a smartwatch, we did not test for differences in the use of this device.

### RQ2: Weekly Use of Different Types of Apps

Of the 725 participants, 660 (91%) reported using one or more smart devices on which an app could be installed (ie, a smartphone, tablet, or smartwatch). Their weekly use of these apps is shown in Table 1. Use of communication apps such as an email app or WhatsApp was common in the total sample: 83.5% (551/660). Social media apps such as Facebook, Instagram, and Twitter were used weekly by little more than half of the total sample: 56.7% (374/660). Of the normal hearing participants, 63.3% (198/313) made weekly use of social media apps compared with 50.7% (176/347) of the adults with hearing impairment. After adjustment for age, a statistically significant difference was found between the groups, with adults with hearing impairment 33% less likely to use social media apps (OR 0.67, 95% CI 0.48-0.92; *P*=.02; Table 2). This was the only type of app for which statistically significant differences in weekly use were found.

### RQ3: Use of Social Media

Of 722 participants, 625 (86.6%) reported using social media. Use of social media did not differ between adults with normal hearing and those with hearing impairment (age-adjusted OR 0.90, 95% CI 0.57-1.4; *P*=.65; Table 2).

We also looked at the frequency with which social media was used. Most of the participants (551/627, 87.9%) used social media daily or multiple times a day. Of note, of the 627 participants, 2 (0.3%) had a missing score on use of social media, but they answered the question about frequency. The age-adjusted cumulative OR for frequency of use of social media for adults with hearing impairment compared with normal hearing adults was 0.91 (95% CI 0.67-1.2; *P*=.56), showing that there was no statistically significant difference in the frequency of social media use between the groups.

### RQ4: Reasons for Using Social Media

Table 3 shows the distribution of the answers for agreements with reasons for using social media. Most of the participants agreed with the statements that they use social media to stay in touch with family members (213/624, 34.1%, score=10-11; 193/624, 30.9%, score=8-9) and with friends (199/625, 31.8%, score=10-11; 227/624, 36.4%, score=8-9). The statement that they use social media to stay in touch with acquaintances was also agreed with by most of the participants, with 31.8% (199/625) scoring 10-11 and 43.4% (271/625) scoring 7-9. Use of social media to expand their work-related network, perform their work, or file complaints and problems was fully disagreed with by most of the participants; 42.1% (257/611), 45.9% (281/611), and 43.5% (263/605), respectively, scored 1 on this question.

**Table 3:** Reasons to use social media: descriptive outcomes for the answers given on an 11-point Likert scale, divided over 3 percentile groups representing the lowest, middle, and highest levels of agreement for that statement in approximate tertiles.^a^

I use social media to...	Lowest-level agreement			Middle-level agreement			Highest-level agreement	
	Value, n (%)	Value, mean (SD; range)	Value, n (%)		Value, mean (SD; range)	Value, n (%)		Value, mean (SD; range)
Stay in touch with family members	218 (35.9)	4.1 (2.4; 1-7)	193 (31.8)		8.6 (0.5; 8-9)	213 (35.1)		10.8 (0.4; 10-11)
Stay in touch with friends	199 (31.8)	4.6 (2.3; 1-7)	227 (36.3)		8.6 (0.5; 8-9)	199 (31.8)		10.7 (0.5; 10-11)
Stay in touch with acquaintances	186 (30.5)	4.2 (2.1; 1-6)	271 (44.4)		8.3 (0.8; 7-9)	153 (25.1)		10.7 (0.5; 10-11)
Stay in touch with colleagues or peers	202 (33.2)	1.6 (1.0; 1-4)	169 (27.8)		6.2 (0.6; 5-7)	237 (39)		9.2 (1.1; 8-11)
Share experiences, videos, or photos	173 (28)	2.2 (1.3; 1-5)	251 (40.7)		7.0 (0.8; 6-8)	193 (31.1)		9.8 (0.9; 9-11)
View experiences, videos, or photos	207 (33.6)	3.8 (2.1; 1-6)	173 (28.1)		7.6 (0.5; 7-8)	236 (38.3)		9.8 (0.9; 9-11)
Expand my work-related network	257 (42.1)	1.0 (0.0; 1-1)	130 (21.3)		3.1 (1.2; 2-5)	224 (36.7)		7.7 (1.6; 6-11)
Perform my work	281 (46)	1.0 (0.0; 1-1)	112 (18.3)		3.2 (1.1; 2-5)	218 (35.7)		7.8 (1.8; 6-11)
Gain new knowledge	204 (33.7)	1.6 (1.0; 1-4)	234 (38.6)		6.9 (1.0; 5-8)	168 (27.7)		9.7 (0.8; 9-11)
File complaints and problems with the government or businesses	263 (43.5)	1.0 (0.0; 1-1)	142 (23.5)		3.1 (1.1; 2-5)	200 (33.1)		7.5 (1.6; 6-11)

^a^The numbers in each percentile group can be dissimilar because the group cannot be broken up within a specific Likert score. The groups are therefore an approximation of percentile groups based on thirds of the sample.

The analyses of differences between participants with normal hearing and those with impaired hearing showed 3 statistically significant results (Table 4). Ordinal regression analysis revealed that participants with hearing impairment were more likely to score a higher level of agreement, compared with the lowest level, with the statement that they use social media to stay in touch with family members (OR 1.5, 95% CI 1.2-2.1; *P*=.003) and, after correcting for age, to stay in touch with friends (OR 1.4, 95% CI 1.0-1.8; *P*=.046). Multinomial regression analysis, in which ORs are estimated separately for the levels of agreement, showed that participants with hearing impairment were also more likely to have the highest level of agreement (score 6-11 on the agreement scale) with using social media to perform their work (age-adjusted OR 1.5, 95% CI 1.04-2.2; *P*=.03). However, the comparison for the middle level of agreement (score 2-5) did not show a statistically significant relationship (age-adjusted OR 0.98, 95% CI 0.62-1.5; *P*=.93), meaning that normal hearing participants and those with impaired hearing were equally likely to score 2-5 on this statement compared with scoring 1.

**Table 4:** Cumulative odds ratios (ORs) for being in a higher percentile of agreeing with reasons for using social media for adults with hearing impairment compared with normal hearing adults; results from (multiple) ordinal logistic regression analysis.

I use social media to...			Crude model				Age-adjusted model^a^		
			OR (95% CI)		*P* value		OR (95% CI)		*P* value
Stay in touch with family members			1.55 (1.16-2.07)		.003		N/A^b^		N/A
Stay in touch with friends			1.25 (0.94-1.67)		.13		1.35 (1.01-1.82)		.046
Stay in touch with acquaintances			1.14 (0.85-1.53)		.34		N/A		N/A
Stay in touch with colleagues or peers			1.03 (0.77-1.38)		.84		1.28 (0.94-1.73)		.11
Expand my work-related network			0.85 (0.63-1.14)		.27		1.03 (0.76-1.41)		.83
**Perform my work (reference category is lowest-level agreement^c^)**									
	Middle-level agreement	0.83 (0.54-1.29)		.41		0.98 (0.62-1.55)		.93	
	Highest-level agreement	1.34 (0.94-1.91)		.11		1.51 (1.04-2.18)		.03	
Share experiences, videos, or photos			1.14 (0.85-1.53)		.37		N/A		N/A
View experiences, videos, or photos			0.95 (0.71-1.27)		.71		1.05 (0.77-1.42)		.77
Gain new knowledge			1.06 (0.79-1.43)		.68		N/A		N/A
File complaints and problems with the government or businesses			1.17 (0.87-1.58)		.29		1.06 (0.78-1.44)		.71

^a^Age in quartiles: Q1: 29-49 years; Q2: 50-59 years; Q3: 60-66 years; Q4: 67-81 years.

^b^N/A: not applicable; no age adjustment necessary.

^c^Multinomial logistic regression analysis.

### RQ5: Experienced Benefits From Using Social Media

Table 1 shows the benefits that participants experienced from using social media. Almost a third (202/628, 32.2%) of the whole group agreed that using social media had given them closer or more intense family ties. Approximately 1 in 4 (161/628, 25.6%) had gained closer or more intense friendships from using social media. New friendships were found by only 11.5% (72/628) of the whole group, and gaining acquaintances was agreed with at a slightly higher rate (114/628, 18.2%). There was also a substantial percentage of participants who had gained little or nothing from social media: 29.3% (184/628).

Logistic regression analysis showed no differences between adults with hearing impairment and those with normal hearing for the experienced benefits from social media use (Table 2).

## Discussion

### Principal Findings

Adults with hearing impairment and normal hearing adults did not differ in either weekly use of a smartphone or weekly use of a tablet. Adults with hearing impairment were less likely to make weekly use of social media apps on a smartphone, tablet, or smartwatch, but they were not less likely to use social media on all types of devices (including a desktop computer or laptop). Compared with normal hearing adults, adults with hearing impairment were more likely to agree with the statements that they use social media to stay in touch with family members and to stay in touch with their friends. Furthermore, participants with hearing impairment were more likely to be in the group that very much agrees with the statement that they use social media to perform their work. The experienced benefits from social media did not differ between adults with hearing impairment and those with normal hearing.

### RQ1: Weekly Use of a Smart Device

Among all NL-SH participants, 84.4% (612/725) made weekly use of a smartphone. This percentage is comparable with the 85% of households in the Netherlands that owned a smartphone in 2017 [34], although it should be noted that our study concerned use of a device and not ownership. After correction for age, we found no differences between adults with normal hearing and those with hearing impairment. Our results therefore are not consistent with those of a 2018 UK survey [21]. This survey found that the rates of smartphone ownership were 53% in people with self-reported hearing impairment and 81% in people who reported having no disabilities. However, 60% of the survey respondents with a self-reported hearing impairment were aged >65 years compared with only 16% of the nondisabled respondents. Although older adults in the United Kingdom are catching up on smartphone use, in 2018 they were still behind younger groups [35]. The results of the UK survey therefore likely reflect the differences in age between both groups.

A tablet is used on a weekly basis by 57.9% (420/725) of all NL-SH participants. In 2017, 66% of Dutch households owned a tablet, which is somewhat higher than in our sample [34]. We found no differences in the use of these devices between adults with normal hearing and those with hearing impairment. No comparisons can be made with other literature because this difference has not been studied before.

### RQ2: Weekly Use of Different Types of Apps

Of the 91% (660/725) of the NL-SH participants who use a smart device, 83.5% (551/660) make weekly use of a communication app such as an email app or WhatsApp. Statistics Netherlands found that 80% of the Dutch general population aged ≥12 years used direct messaging (mostly WhatsApp) in 2017 [34]. This proportion is comparable with the one we found for the weekly use of a communication app. In contrast, Ipsos found a slightly lower percentage for Dutch smartphone users aged 18-64 years [36]. They reported that 68% had used an app to communicate with people in the previous 30 days (data collected in 2017). Use of the following types of apps can be compared with the Ipsos results: weather: NL-SH 69.1% (456/660), Ipsos 54%; news: NL-SH 68.6% (423/660), Ipsos 44%; financial: NL-SH 33.6% (222/660), Ipsos 51%; music and podcasts: NL-SH 26.5% (175/660), Ipsos (listen to music) 37%; and fitness (tracking): NL-SH 8.9% (59/660), Ipsos 20%. Overall, the percentages from the Ipsos report deviate from the ones we found, likely due to differences in the composition of the samples. The mean age of the Ipsos Dutch sample was 40.4 (SD not reported) years (NL-SH 57.7, SD 11.4 years), with 50% of the participants being women (NL-SH: 442/725, 61%) and 78% reporting to be working (NL-SH: 331/725, 45.7%).

We found the rate of overall weekly use of medical and health apps, other than fitness tracking apps, to be 12.4% (82/660). Overall use of these apps among the Dutch population is not known, but in February 2019 the most used nonfitness apps were diet-tracking apps (10.8%), first-aid apps (9.2%), and sleep-tracking apps (8.1%) [37]. These percentages seem to be somewhat higher than those in the NL-SH sample, but this could also be because most of our measurements were performed in 2017. It was only for use of social media apps that we found a difference between adults with normal hearing and those with hearing impairment. After adjustment for age, the adults with hearing impairment had a 33% (OR 0.67, 95% CI 0.48-0.92) lower odds of using social media apps. Another study looked at the use of apps by adults with hearing impairment. A 2016 US survey asked people with self-reported hearing disabilities which features or functions they use on their smartphone and mirrored this with results for all American adults [22]. In the survey, the use of social media apps seems to be lower in people with a self-reported hearing disability (64% vs 75% of all American adults), but no statistical testing was done, nor was an adjustment made for potential age differences. Therefore, it is uncertain if these results agree with those of our study.

It therefore seems that, apart from the use of social media apps, no notable differences exist in the type of apps that adults with hearing impairment use compared with normal hearing adults, but other research should confirm these results.

### RQ3: Use of Social Media

Of the NL-SH participants, 86.6% (625/722) used social media on any type of device. Again, this is comparable with the general Dutch population: 85% used social media in 2017 [34]. Most of the NL-SH participants who use social media do this daily or multiple times a day (551/627, 87.9%). This outcome is not available for the general Dutch population, but there is a marketing report describing 72% of WhatsApp users (WhatsApp is the most frequently used social media app in the Netherlands) using this app on a daily basis in 2017 [38]. This is lower than the 87.9% (551/627) we find for social media overall, but this difference is likely caused by this percentage only covering a single social media platform that is also primarily used on a smart device.

Despite the lower weekly use of social media apps by adults with hearing impairment, overall social media use did not differ between adults with normal hearing and those with hearing impairment. A reason for this could be that, in the question on the use of apps, we made a distinction between communication apps (email and direct messaging apps such as WhatsApp) and social media apps (eg, Facebook, Instagram, and Twitter; Textbox 1). The latter addresses use of social networking sites. In the question about overall social media use, direct messaging and social networking sites were taken together. The addition of direct messaging to this question may have obfuscated the difference in use of social networking sites. Another explanation could lie in device preferences. Many social media platforms also run in a web browser. Perhaps adults with hearing impairment have a preference for approaching these social media through a web browser on a laptop or PC because of their larger screens. It is also possible that they prefer to use these devices because they can easily be set up with a speaker. From the hearing test results we know that 71.9% (276/384) of the participants with hearing impairment performed this test with speakers as opposed to using a headphone, whereas this rate was 37.9% (129/340; the type of transducer used was missing for 1 participant) in normal hearing participants. It should also be taken into account that different social media platforms attract users with different characteristics [39]. As we did not ask about distinct social media platforms, we could not compare their specific use. The results could therefore also imply that adults with hearing impairment make less use of social media platforms that are mainly used as an app, for example, Instagram. A final reason for the divergence could be that the use of apps concerned weekly use, whereas use of social media did not specify frequency of use.

We also did not find differences in the frequency of overall social media use. The sparse literature on the latter outcome shows contradictory results and focuses on young people who were born deaf or hard of hearing or became deaf or hard of hearing at a young age [40]. This group is not representative of the participants with hearing impairment in the NL-SH, most of whom have age-related hearing loss. There are important differences in proficiency in reading and writing and in their self-identification between these groups that will likely affect their social media use.

### RQ4: Reasons for Using Social Media

A comprehensive theoretical framework on what people do on social media and why they use social media has not been established. Several motivations [41] and theories [42] have been put forward. The activities we asked about in our reasons for using social media mostly fall within the motivations summed up previously [41]. In line with the literature [41,43] and marketing research [44], we found considerable agreement with using social media to keep in touch with people, especially those in the private social network [44]. This is also confirmed by the fact that 80% of the Dutch people aged ≥12 years use social media for direct messaging. There was also agreement with using social media to connect with colleagues and to create content and consume content, but this was less pronounced. These reasons also concern relationships with other people but are probably less important because the first reason pertains to professional contacts and the latter 2 concern less direct ways of connecting with others. Although 30% of the population of the Netherlands were said to use a professional social network in 2017 [34], daily use of LinkedIn is limited [38]. This confirms the disagreement we found with the statements about social media and work. NL-SH participants also do not agree with filing complaints and problems (with businesses and organizations) using social media. Marketing research in 2017 shows similar outcomes: 9% of Dutch people contacted customer services through WhatsApp, and only 12% of them had a preference for this [45]. There is a major preference for traditional contact through phone and email. Agreement with using social media to gain new knowledge was fairly evenly divided across the participants. The literature also mentions this as a motivation for using social media but gives no clues about its relative importance for using social media [41].

In the comparison between the 2 groups, we found that participants with hearing impairment are more likely to agree with using social media to stay in touch with family members and friends. They were also more likely to have the highest level of agreement with using social media to perform their work compared with the lowest level of agreement. The results of research among young people who are deaf and hard of hearing show that the most frequent motive to use social media is to maintain social contact, although no comparisons were made with normal hearing people [40].

No other studies could be found on use of social media for work purposes among adults with hearing impairment. Thus, we are the first to report that agreement with using social media to keep in contact with family and friends and to perform work is higher among adults with hearing impairment than among normal hearing peers.

### RQ5: Experienced Benefits From Using Social Media

Gains or benefits experienced from using social media are conflated with the reasons to use social media. The finding that almost a third (202/628, 32.2%) of the whole group agreed that using social media had given them closer or more intense family ties and that 25.6% (161/628) had gained closer or more intense friendships ties in with many participants using social media to stay in touch with people close to them. Research in adolescents shows that social media can have a positive effect on their social connectedness and sense of belonging [46]. Positive results on social well-being are also found in adults but only when there is low emotional investment [47].

We found no differences between adults with hearing impairment and those with normal hearing for the experienced benefits from social media use. This suggests that despite our previous finding that participants with hearing impairment are more likely to use social media to stay in touch with family members and friends and to perform their work, they derive benefits from this similar to those derived by normal hearing adults.

### Implications for Clinical Practice and Research

The broad use of smart devices in our sample shows the potential for digitalized hearing health care through specific apps. However, it should be noted that 9% (65/725) of the NL-SH participants did not use a smart device and that this was more prominent in older adults. Of all study participants aged >65 years, 15.7% (182/216) did not use a smart device (results not shown). As most of the people with hearing impairment are aged >65 years, this means that a substantial number of people with hearing impairment access the internet by other means. To ensure equal access, digital hearing health care should not be restricted to apps; it should also be available on desktop computers and laptops. This ties in with the possibility that adults with hearing impairment have a preference to use these latter devices to access social media.

The use of medical and health apps appears low in our sample because only 12.4% (82/660) reported using these apps and their use is apparently higher in the general public. This does not imply that adults with hearing impairment have little interest in apps for hearing health care; rather, it means that more research is needed into their needs and wants. A variety of solutions may be necessary, as was shown in previous research on eHealth alongside the customer journey of older people with hearing loss [48].

We speculated that communication through the internet could be specifically attractive to adults with hearing impairment. Indeed, adults with hearing impairment were more likely to use social media to stay in touch with family members and friends than normal hearing adults. Nonetheless, they were not more likely to report closer or more intense family ties or friendships from using social media. This shows that internet-mediated communication does not have more salience for adults with hearing impairment than other modes of communication. More research is needed on the type of social media that adults with hearing impairment use to stay in touch with close family members and friends and whether the higher use mitigates mental health outcomes as we have put forward.

Finally, participants with hearing impairment were more likely to use social media to perform their work. Work can be challenging for people with hearing loss because of the difficulties in spoken communication. This can result in increased levels of fatigue, stress, loss of productivity, and job loss [49,50]. It would be of interest to study how adults with hearing impairment use social media to perform their work and whether social media use can help to mitigate these adverse effects. Consideration could also be given to using social media for audiological rehabilitation purposes, for instance, providing the option to consult vocational rehabilitation services through direct messaging, but this needs further investigation.

### Strengths and Limitations

The strengths of this study include the large sample, its diversity in age, the comparison between adults with normal hearing and those with hearing impairment, the correction for confounders, and the functional measurement of hearing ability. As all measurements were performed through a questionnaire and a hearing test that ran in a web browser, the study was likely to attract adults who are fairly comfortable with using the internet through a web browser. Nevertheless, the total sample seems representative of the general Dutch population in their use of technology and social media.

Our study includes several limitations. We used existing questionnaires that are used for marketing purposes. We were not able to find any documents showing validity of these questionnaires. Nevertheless, given that they are used in marketing research, we assume that they were developed according to accepted standards and hence valid, although a scientific report on their validity has not been published. We assume that the questions on use of technology and different types of apps were valid. The questions we devised on several aspects of social media use were not tested for validity either. This needs further investigation. In addition, all data were self-reported and could therefore suffer from recall bias, particularly the questions that included frequency of use, as well as from social desirability bias. The actual use of apps and social media on smart devices can be measured by individually tracking their use with software that collects these data and sends them to a research database [51], but this was not feasible in this study. Given that we did not ask about controversial topics with respect to using the internet and social media as well as the rather anonymous nature of filling out a web-based questionnaire, we believe that social desirability bias is of minimal importance in our study. Another limitation is the use of convenience sampling. The NL-SH sample is more highly educated than the general Dutch population, which could hamper generalization. However, the comparisons between adults with hearing impairment and those with normal hearing are still valid because they were performed within this sample and no influence of education level was found on the associations. All analyses were cross-sectional, which does not allow for inferences on causal relationships. Finally, we performed a very large number of statistical tests. The probability of a type 1 error, finding a statistically significant difference when in reality there is no difference, increases with multiple testing. We did not correct for this because we considered our study to be exploratory in nature. The results should be interpreted accordingly.

### Conclusions

The potential for digitalized hearing health care is confirmed. Adults with impaired hearing are not less likely to use smart devices than their normal hearing peers. More research is needed into the needs and wants of adults with hearing impairment for the type of hearing health care solutions they seek.

Adults with hearing impairment agreed more with using social media to stay in touch with family members and friends than normal hearing adults, but this did not result in closer or more intense family ties or friendships. Research is needed on the type of social media that adults with hearing loss use to stay in touch with close family members and friends and to determine whether the higher use of social media mitigates mental health outcomes as we have put forward. Given that participants with hearing impairment are more likely than their normal hearing peers to use social media to perform their work, it would be of interest to study whether and how vocational rehabilitation services for workers with hearing impairment could be implemented on social media platforms as an alternative or supplementary to standard hearing health care. Adults with hearing impairment are less likely to make weekly use of social media apps on a smartphone, tablet, or smartwatch but not less likely to use social media on all types of internet-connected devices. This warrants further research on the types of social media platforms that adults with hearing impairment use and on the type of device on which they prefer to use social media.

## Appendix

Multimedia Appendix 1 Questions from the National Longitudinal Study on Hearing questionnaire used for this study.

## Abbreviations

NHT: National Hearing Test
NL-SH: National Longitudinal Study on Hearing
OR: odds ratio
RQ: research question
SNR: signal-to-noise ratio
SRTn: speech-reception threshold in noise

## References

[1] World Health Organization (2018). Addressing the rising prevalence of hearing loss.

[2] Bainbridge KE, Wallhagen MI (2014). Hearing loss in an aging American population: extent, impact, and management. Annu Rev Public Health.

[3] Homans NC, Metselaar RM, Dingemanse JG, van der Schroeff MP, Brocaar MP, Wieringa MH, de Jong RJ, Hofman A, Goedegebure A (2017). Prevalence of age-related hearing loss, including sex differences, in older adults in a large cohort study. Laryngoscope.

[4] van der Meijden W, Panneman M, Kloet S, Blatter B, Homans N, Goedegebure A (2020). Prevalentie van gehoorverlies in Nederland. VeiligheidNL, Kenniscentrum Letselpreventie.

[5] Hunsaker A, Hargittai E (2018). A review of Internet use among older adults. New Media Society.

[6] (2020). Ageing Europe - statistics on social life and opinions. Eurostat.

[7] Paglialonga A, Nielsen A, Ingo E, Barr C, Laplante-Lévesque A (2018). eHealth and the hearing aid adult patient journey: a state-of-the-art review. Biomed Eng Online.

[8] Paglialonga A, Tognola G, Pinciroli F (2015). Apps for hearing healthcare. Stud Health Technol Inform.

[9] Meijerink JF, Pronk M, Lissenberg-Witte BI, Jansen V, Kramer SE (2020). Effectiveness of a web-based SUpport PRogram (SUPR) for hearing aid users aged 50+: two-arm, cluster randomized controlled trial. J Med Internet Res.

[10] Pronk M, Besser J, Smits C, Feenstra-Kikken V, van Beek H, Polleunis C, Kramer SE (2020). Rationale, theoretical underpinnings, and design of HEAR-aware: providing adults with hearing loss with tailored support to self-manage their hearing problems via a smartphone app, as an alternative to hearing aids. Am J Audiol.

[11] Saunders GH, Roughley A (2021). Audiology in the time of COVID-19: practices and opinions of audiologists in the UK. Int J Audiol.

[12] Swanepoel D, Hall J (2020). Making audiology work during COVID-19 and beyond. Hearing J.

[13] Shukla A, Harper M, Pedersen E, Goman A, Suen JJ, Price C, Applebaum J, Hoyer M, Lin FR, Reed NS (2020). Hearing Loss, Loneliness, and Social Isolation: A Systematic Review. Otolaryngol Head Neck Surg.

[14] Nachtegaal J, Smit JH, Smits C, Bezemer PD, van Beek JH, Festen JM, Kramer SE (2009). The association between hearing status and psychosocial health before the age of 70 years: results from an internet-based national survey on hearing. Ear Hear.

[15] Stam M, Smit JH, Twisk JW, Lemke U, Smits C, Festen JM, Kramer SE (2016). Change in psychosocial health status over 5 years in relation to adults' hearing ability in noise. Ear Hear.

[16] Blazer DG, Tucci DL (2019). Hearing loss and psychiatric disorders: a review. Psychol Med.

[17] Chen E, Wood D, Ysseldyk R (2021). Online social networking and mental health among older adults: a scoping review. Can J Aging.

[18] Gonsalves C, Pichora-Fuller MK (2008). The effect of hearing loss and hearing aids on the use of information and communication technologies by community-living older adults. Can J Aging.

[19] Henshaw H, Clark DP, Kang S, Ferguson MA (2012). Computer skills and internet use in adults aged 50-74 years: influence of hearing difficulties. J Med Internet Res.

[20] Thorén ES, Oberg M, Wänström G, Andersson G, Lunner T (2013). Internet access and use in adults with hearing loss. J Med Internet Res.

[21] (2019). Disabled users access to and use of communication devices and services - Research summary: all disabled people. Ofcom.

[22] Morris J, Sweatman W, Jones M (2017). Smartphone use and activities by people with disabilities: user survey 2016. J Technol Persons Disab.

[23] Shoham S, Heber M (2012). Characteristics of a virtual community for individuals who are d/deaf and hard of hearing. Am Ann Deaf.

[24] Saxena RC, Lehmann AE, Hight AE, Darrow K, Remenschneider A, Kozin ED, Lee DJ (2015). Social media utilization in the cochlear implant community. J Am Acad Audiol.

[25] Choudhury M, Dinger Z, Fichera E (2017). The utilization of social media in the hearing aid community. Am J Audiol.

[26] Crowson MG, Tucci DL, Kaylie D (2018). Hearing loss on social media: who is winning hearts and minds?. Laryngoscope.

[27] Bahng J, Lee CH (2020). Topic modeling for analyzing patients' perceptions and concerns of hearing loss on social Q and A sites: incorporating patients' perspective. Int J Environ Res Public Health.

[28] Kožuh I, Debevc M (2019). The utilisation of social media among users with hearing loss: an analysis of Facebook communities. Univ Access Inf Soc.

[29] National Hearing Test.

[30] NL-SH study website for the general public and participants. National Longitudinal Study on Hearing.

[31] Kramer SE, Kapteyn TS, Festen JM (1998). The self-reported handicapping effect of hearing disabilities. Audiology.

[32] Smits C, Kapteyn TS, Houtgast T (2004). Development and validation of an automatic speech-in-noise screening test by telephone. Int J Audiol.

[33] Smits C, Merkus P, Houtgast T (2006). How we do it: the Dutch functional hearing-screening tests by telephone and internet. Clin Otolaryngol.

[34] Arends-Tóth J (2019). ICT-gebruik van huishoudens en personen [ICT use of households and persons]. ICT, kennis en economie.

[35] (2018). Smartphone penetration by age group (2012-18). Deloitte.

[36] Ledbury Celine, Woolhead Laura, Thomas Ffion, Carpenter Reece, Stott Hannah (2017). Something for everyone. Why the growth of mobile apps is good news for brands. ipsos MORI.

[37] (2019). Monitor uw eigen gezondheid met deze tien apps. Patientline Whitepaper.

[38] Van der Veer N, Boekee S, Hoekstra H, Peters O (2018). Nationale Social Media Onderzoek 2018 [National Social Media Study 2018]. Newcom Research and Consultancy.

[39] Blank G, Lutz C (2017). Representativeness of social media in Great Britain: investigating Facebook, LinkedIn, Twitter, Pinterest, Google+, and Instagram. Am Behav Sci.

[40] Kožuh I, Debevc M, Dey N, Babo R, Ashour A, Bhatnagar V, Bouhlel M (2018). Challenges in social media use among deaf and hard of hearing people. Social Networks Science: Design, Implementation, Security, and Challenges.

[41] Hoffman DL, Novak TP (2020). Why do people use social media? Empirical findings and a new theoretical framework for social media goal pursuit. SSRN J.

[42] Ngai EW, Tao SS, Moon KK (2015). Social media research: theories, constructs, and conceptual frameworks. Int J Inform Manag.

[43] Perugini ML, Solano AC (2020). Normal and maladaptive personality traits as predictors of motives for social media use and its effects on well-being. Psychol Rep.

[44] Heimlich R (2011). Using social media to keep in touch. Pew Research Center.

[45] van Manen T (2017). Onderzoek: het gebruik van internet en social media in Nederland [Research: the use of internet and social media in the Netherlands]. Marketingfacts.

[46] Allen KA, Ryan T, Gray DL, McInerney DM, Waters L (2020). Social media use and social connectedness in adolescents: the positives and the potential pitfalls. Aus Edu Develop Psychol.

[47] Bekalu MA, McCloud RF, Viswanath K (2019). Association of social media use with social well-being, positive mental health, and self-rated health: disentangling routine use from emotional connection to use. Health Educ Behav.

[48] Nielsen AC, Rotger-Griful S, Kanstrup AM, Laplante-Lévesque A (2018). User-innovated eHealth solutions for service delivery to older persons with hearing impairment. Am J Audiol.

[49] Nachtegaal J, Festen JM, Kramer SE (2012). Hearing ability in working life and its relationship with sick leave and self-reported work productivity. Ear Hear.

[50] Nachtegaal J, Kuik DJ, Anema JR, Goverts ST, Festen JM, Kramer SE (2009). Hearing status, need for recovery after work, and psychosocial work characteristics: results from an internet-based national survey on hearing. Int J Audiol.

[51] Araujo T, Wonneberger A, Neijens P, de Vreese C (2017). How much time do you spend online? Understanding and improving the accuracy of self-reported measures of internet use. Commun Methods Measures.

