# Supplementary material for: A Comparison of the Use of Smart Devices, Apps, and Social Media Between Adults With and Without Hearing Impairment: Cross-sectional Web-Based Study
Source: J Med Internet Res. 2021 Dec 20;23(12):e27599. doi: 10.2196/27599 (PMC8726052; doi:10.2196/27599)
Supplement: Multimedia Appendix 1 [file jmir_v23i12e27599_app1.pdf]

Questions from the 10-year measurement round of the National Longitudinal Study on Hearing which were used in the analyses for the study "A Comparison of the Use of Smart Devices, Apps, and Social Media Between Adults With and Without Hearing Impairment"

1. How would you describe your hearing ability?
   - o I have normal hearing
   - o I am hearing impaired, I have conductive hearing loss
   - o I am hearing impaired, I have sensorineural hearing loss
   - o I am hearing impaired, I have mixed hearing loss
   - o I am hearing impaired, I have Meniere disease
   - o I am hearing impaired but I don't know the type of hearing loss I have
   - o I am single sided deaf but normally hearing in the other ear
   - o I am singled sided deaf, and hearing impaired in the other ear

If 1 = normal hearing → 3

2. Do you use a hearing aid?
   - o Yes
   - o No

3. Are you male or female?
   - o Male
   - o Female

4. What is your age?
   ………… years

5. Is Dutch your native language?
   - o Yes
   - o No

6. What is the highest educational level that you completed?
   - o Did not finish elementary school
   - o Elementary school
   - o Lower-level secondary education
   - o Middle-level secondary education
   - o Higher-level secondary education - 5 years
   - o Higher-level secondary education - 6 years
   - o Middle-level applied education (junior college)
   - o University of applied sciences (college)
   - o Research University (Master's degree)
   - o Post academic degree (e.g. PhD)

7. Which of these devices do you use at least once a week?
   - ☐ Traditional cell phone
   - ☐ Smartphone (Cell phone with many functions like a small computer. Typically has a large display, iPhone or similar from another brand)
   - ☐ Traditional wrist watch
   - ☐ Smartwatch (A watch with many functions like a computer. Apple watch or similar)
   - ☐ Camera/video camera
   - ☐ Laptop or notebook computer
   - ☐ Fitness watch (watch that is worn to for instance measure the heartbeat or to count steps. Fitbit or similar from other brand)
   - ☐ Headset/headphones
   - ☐ Landline phone
   - ☐ Radio or stereo system
   - ☐ Tablet computer (iPad or tablet of another brand)
   - ☐ TV
   - ☐ MP3 player (iPod or similar from another brand)
   - ☐ Other, namely …….
   - ☐ None of the above

If 8 = Smartphone and/or Smartwatch and/or Tablet computer:

8. Which types of apps do you use at least once a week on your current smartphone, tablet, or smartwatch?
   - ☐ Weather
   - ☐ News
   - ☐ Finances (mobile banking, stock exchange, etc.)
   - ☐ Navigation
   - ☐ Remote control (TV, stereo system, etc.)
   - ☐ Fitness
   - ☐ Communication (E-mail, whatsapp, WeChat, etc)
   - ☐ Medical/health
   - ☐ Social media (Facebook, Instagram, Twitter, etc)
   - ☐ Music & Podcasts
   - ☐ Other, namely ……

9. Do you use social media? (E.g. Facebook, Linkedin, Skype, Whatsapp, Twitter, Instagram)
   - o Yes
   - o No

If 9= no → end of questionnaire

10. To what extent do you agree with the following statements? I use social media to……

	**Fully disagree 0**	**1**	**2**	**3**	**4**	**5**	**6**	**7**	**8**	**9**	**Fully agree 10**
Stay in touch with family members	○	○	○	○	○	○	○	○	○	○	○
Stay in touch with acquaintances	○	○	○	○	○	○	○	○	○	○	○
Stay in touch with colleagues or peers	○	○	○	○	○	○	○	○	○	○	○
Share experiences, videos or photos	○	○	○	○	○	○	○	○	○	○	○
View experiences, videos, or photos	○	○	○	○	○	○	○	○	○	○	○
Expand my work-related network	○	○	○	○	○	○	○	○	○	○	○
Perform my work	○	○	○	○	○	○	○	○	○	○	○
Gain new knowledge	○	○	○	○	○	○	○	○	○	○	○
File complaints and problems with the government of businesses	○	○	○	○	○	○	○	○	○	○	○

11. With what frequency do you use social media?
   - ○ Multiple times a day
   - ○ Daily
   - ○ Weekly
   - ○ Monthly
   - ○ A couple of times a year

12. What have you gained from your social media use so far?
   - ☐ New acquaintances
   - ☐ New friendships
   - ☐ Closer/more intense family ties
   - ☐ Closer/more intense friendships
   - ☐ Expanded work-related network
   - ☐ New knowledge about health
   - ☐ I have gained little or nothing from social media
   - ☐ Other, namely….
